# The Impact of Lab4 Probiotic Supplementation in a 90-Day Study in Wistar Rats

**DOI:** 10.3389/fnut.2021.778289

**Published:** 2021-11-25

**Authors:** Thomas S. Webberley, Giulia Masetti, Laura M. Baker, Jordanna Dally, Timothy R. Hughes, Julian R. Marchesi, Alison A. Jack, Sue F. Plummer, Guru Ramanathan, Paul D. Facey, Daryn R. Michael

**Affiliations:** ^1^Cultech Limited, Port Talbot, United Kingdom; ^2^Swansea University Medical School, Swansea University, Swansea, United Kingdom; ^3^Division of Infection and Immunity, School of Medicine, Cardiff University, Cardiff, United Kingdom; ^4^Division of Digestive Diseases, Department of Metabolism, Digestion and Reproduction, Faculty of Medicine, Imperial College London, London, United Kingdom; ^5^Pharmacology based Clinical Trials, Pennington Biomedical Research Centre, Baton Rouge, LA, United States

**Keywords:** probiotic, inflammation, SCFA, cholesterol, bile, cardiovascular

## Abstract

The anti-inflammatory and cholesterol lowering capabilities of probiotic bacteria highlight them as potential prophylactics against chronic inflammatory diseases, particularly cardiovascular disease. Previous studies *in silico, in vitro*, and *in vivo* suggest that the Lab4 probiotic consortium may harbour such capabilities and in the current study, we assessed plasma levels of cytokines/chemokines, short chain fatty acids and lipids and faecal levels of bile acids in a subpopulation of healthy Wistar rats included in 90-day repeat dose oral toxicity study. In the rats receiving Lab4, circulating levels of pro-inflammatory interleukin-6, tumour necrosis factor-α and keratinocyte chemoattractant/growth regulated oncogene were significantly lower compared to the control group demonstrating a systemic anti-inflammatory effect. These changes occurred alongside significant reductions in plasma low density lipoprotein cholesterol and increases in faecal bile acid excretion implying the ability to lower circulating cholesterol *via* the deconjugation of intestinal bile acids. Correlative analysis identified significant associations between plasma tumour necrosis factor-α and the plasma total cholesterol:high density lipoprotein cholesterol ratio and faecal levels of bifidobacteria in the Lab4 rats. Together, these data highlight Lab4 supplementation as a holistic approach to CVD prevention and encourages further studies in humans.

## Introduction

The trillions of microorganisms residing in the human gut (the gut microbiota) are intrinsically linked to the well-being of the host (1). Perturbations in the microbiota have been linked with a range of diseases although it is often unclear if this is a causative factor or an epiphenomenon and stabilization (2). The modulation of the gut microbiota by dietary means is being explored as a means of preserving and rescuing health and attention is turning to the potential role for probiotic organisms that are defined as “live microorganisms that, when administered in adequate amounts, confer a health benefit on the host” (3).

Interest in the anti-inflammatory properties of probiotics is growing particularly due to the potential benefits within the context of chronic inflammatory diseases (CID) where beneficial effects have been observed during rheumatoid arthritis, inflammatory bowel disease and obesity (to name but a few) (4, 5). A CID of particular concern is cardiovascular disease (CVD) that is a progressive inflammatory disease of the vasculature driven by hypercholesterolemia and is responsible for more than 30 % of all global mortalities (6). A recent meta-analysis in high-risk subjects (with hypertension, excess body weight or hypercholesterolemia) concluded that probiotics were particularly effective for the reduction of low density lipoprotein-cholesterol (LDL-C) (7). It is now understood that elevated LDL-C can be a disease risk even in individuals with no history of CVD or any of its comorbidities (8) and that even marginal reductions in LDL-C profoundly lessen risk (9). Mechanistically, the anti-inflammatory capabilities of probiotics has been attributed to, at least in part, the generation of anti-inflammatory short chain fatty acids (SCFA) such as acetate and butyrate (5). The principle mechanism of cholesterol-lowering is related to inherent bile salt hydrolase (BSH) enzymes that deconjugate bile acids in the intestinal lumen rendering them less readily reabsorbed for recycling in the liver (10). The deconjugated bile acids are excreted from the body creating a bile acid deficit in the host that is counteracted by increased hepatic bile synthesis that utilises and therefore reduces circulatory cholesterol levels (11).

The Lab4 probiotic consortium comprises *Bifidobacterium bifidum, Bifidobacterium animalis* subsp. *lactis* and two strains of *Lactobacillus acidophilus* and anti-inflammatory capability has been observed in a previous study where blood mononuclear cells collected from Lab4 supplemented participants showed impaired secretion of interleukin (IL)-6 and tumour necrosis factor (TNF)-alpha *ex vivo* (12). Assessment *in silico* of genomic composition identified genes encoding enzymes involved in the generation of SCFAs in all Lab4 strains (13). The cholesterol-lowering potential of Lab4 is better characterised and evidenced by (i) the ability to reduce both circulating LDL-C levels and bodyweight in hypercholesterolemic overweight and obese adults (14) and (ii) ameliorate the impact of high fat diet (HFD) induced elevations in plasma LDL-C and body weight in wild-type C57BL/6J mice (15). In atherosclerosis prone mice receiving a HFD, Lab4 contributed to the maintenance of plasma LDL-C levels and the inhibition of atherosclerotic plaque (fatty deposit) formation in the vasculature (16). All Lab4 organisms possess the gene sequence encoding the BSH enzyme choloylglycine hydrolase (13) and BSH activity has been observed during both assessments *in vitro* and *in vivo* (17). Studies *in vitro* show that the Lab4 consortium can assimilate cholesterol and regulate cholesterol transport across the intestinal epithelium (17).

This study details the impact of Lab4 on traditional CVD biomarkers (plasma cytokines/chemokines and lipids), plasma SCFAs and faecal bile acid profiles following a 90-day repeat feeding study in healthy wild-type Wistar rats.

## Materials and Methods

### Animal Maintenance and Probiotic Administration

The study was performed within the test facility of INTOX Pvt. Ltd. (Maharashtra, India) in compliance with the OECD Principles of Good Laboratory Practise [Organisation for Economic Co-operation and Development (OECD), 1998]. The animals in this study represent a subpopulation of a repeated dose 90-day oral toxicity study (13). Two groups of 10 male Wistar rats [6–7 weeks old, purchased from Vivo Bio Tech Ltd (Telangana, India)] were housed in pathogen-free ventilated cages (2 rats per cage) in a light- and temperature-controlled facility (12 h light, 12 h dark, 19–25 °C) and received a daily gavage of either the Lab4 probiotic comprising *Lactobacillus acidophilus* CUL21 (NCIMB 30156), *Lactobacillus acidophilus* CUL60 (NCIMB 30157), *Bifidobacterium bifidum* CUL20 (NCIMB 30153) and *Bifidobacterium animalis subsp. lactis* CUL34 (NCIMB 30172) at 3.0 x 10^10^ colony forming units (cfu) in 10 mL phosphate buffered saline (PBS, Lab4 group) or a placebo comprising 10 mL sterile PBS (Control group) for 90 days. Throughout the study, rats were given access *ad libitum* to a standard diet [65 % carbohydrate, 24 % protein, 11 % fat (Altromin 1320, Spezialfutter GmbH & Co. KG, Germany)] and sterilised water in order to conform with animal welfare requirements. Body weight and food consumption were monitored weekly. At the end of the study, rats were sacrificed by exsanguination under CO_2_ asphyxiation.

### Blood Plasma Analysis

Overnight fasted blood was collected into heparinised tubes at day 90. Plasma was extracted *via* centrifugation (1,000 x g, 5 min) and stored at −80 °C pending further analysis. Plasma concentrations of IL-4, IL-6, IL-10, IL-13, TNF-α, interferon-γ (IFN-γ) and keratinocyte chemoattractant/growth regulated oncogene (KC/GRO) were measured by the Central Biotechnology Service (CBS; Cardiff University, UK) using the VPLEX pro-inflammatory panel 1 rat kit (Meso Scale Discovery, Maryland, USA). Total cholesterol (TC), high-density lipoprotein cholesterol (HDL-C) and low-density lipoprotein cholesterol (LDL-C) and triglyceride (TG) concentrations were measured using Cholesterol and Triglyceride assay kits (ABCAM, Cambridge, UK) in accordance with the manufacturer's instructions. Total plasma bile acid levels were measured using the Xpand Plus Clinical Chemistry System (Siemens Healthcare Diagnostics Inc. Newark, U.S.A.) in accordance with the manufacturer's instructions. SCFA were measured using targeted gas chromatography and mass spectroscopy according to Moreau et al. (18).

### Faecal Bile Acid Analysis

Faecal samples collected at day 90 were measured for bile acid content using ultra performance liquid chromatography-mass spectrometry (UPLC-MS), as previously described (19). Faecal pellets were lyophilized for 48 h (VirTis Benchtop BTP 8ZL freeze dryer, BPS FUK), weighed and then homogenised in a mixture of water, acetonitrile and 2-propanol (2:1:1 volumes) using a Biospec bead beater with 1.0 mm Zirconia beads. The homogenates were centrifuged at 16,000 × g for 20 min and the supernatant filtered through a 0.45 μm membrane (Costar, Corning, UK). Bile acid analysis was performed on an ACQUITY UPLC (Waters Ltd, Elstree, UK) coupled to a Xevo G2 Q-ToF mass spectrometer equipped with an electrospray ionisation source operating in negative ion mode and bile acid standards [55 bile acid standards including 36 non-conjugated, 12 conjugated with taurine, seven conjugated with glycine (Steraloids, Newport, RI)] were used to determine the chromatographic retention times. MassLynx software 4.1 was used for data acquisition. Relative faecal bile acid intensities were normalised to the faecal pellet dry weight. Principle component analysis (PCA) was carried out using SIMCA v14.1.0.2047 (MKS Umetrics, Umeå, Sweden) and heat-maps were generated in R package (Version 3.1.3).

### Analysis of Faecal Lactobacilli and Bifidobacteria

Faecal samples were assessed for viable numbers of lactobacilli and bifidobacteria during the study of Baker et al. (13). Briefly, faeces were collected after 90 days intervention and 10-fold dilution series were set up in Maximum Recovery Diluent (MRD). Lactobacilli and bifidobacteria were plated on DeMan Rogosa Sharpe (MRS) medium or MRSX [MRS containing lithium chloride (1 g/L), sodium propionate (1.5 g/L) and L-cysteine hydrochloride (0.25 g/L)] respectively and incubated anaerobically (10 % carbon dioxide, 10 % hydrogen and 80 % nitrogen) at 37 °C. Bacterial species was confirmed by Gram staining, colony morphology and by analytical profile index (API, BioMerieux, Marcy-l'Étoile, France). Viable counts were recorded as the numbers of log_10_ colony forming units (cfus) per gram of sample.

### Statistical Analysis

All data are presented as mean ± standard deviation (SD) of the assigned number of rats. All data were assessed for homogeneity (Levene's test) and normality (Shapiro-Wilk test or inspection of Q-Q plots). For data that were non-normally distributed, log or Box-Cox transformations were performed. For single comparisons, values of *p* were determined using an independent *t*-test (where data were normally distributed) or Wilcoxon-Mann test for non-parametric data. For multiple comparisons, values of *p* were determined using one-way analysis of variance (ANOVA) with Dunnett's T3 *post-hoc* analysis or, where data were not normally distributed after log or Box-Cox transformation, Kruskal-Wallis followed by Dunn's test with Bonferroni correction. All statistical tests were performed using GraphPad 7.0 (Prism). Where sufficient pairwise data was available, correlations were performed with a Spearman's rank coefficient analysis in R package (Version 3.1.3). Differences were considered significant when *p* < 0.05.

## Results

### Plasma Inflammatory Markers

Supplementation with the Lab4 probiotic resulted in a significant 37.05 % reduction in plasma IL-6 (*p* = 0.026, Table 1A), a significant 42.92 % reduction in TNF-α (*p* = 0.005) and a significant 38.53 % reduction in KC/GRO (*p* = 0.036) compared to levels in the control. No changes were observed for the other inflammatory markers tested.

**Table 1:** Plasma levels of inflammatory markers and lipids and faecal bile acid content in male Wistar rats after 90 days supplementation with Lab4.

	**Control**	**Lab4**
***A. Plasma inflammatory markers (pg/mL):***		
Interleukin-4	1.69 ± 0.09	1.67 ± 0.16
Interleukin-6	33.72 ± 6.03	21.23 ± 9.75^*^
Interleukin-10	9.27 ± 1.45	8.55 ± 2.05
Interleukin-13	5.71 ± 1.38	5.57 ± 0.71
Tumour necrosis factor-α	4.66 ± 1.76	2.66 ± 0.54^**^
Interferon-γ	1.90 ± 0.40	1.66 ± 0.27
Keratinocyte chemoattractant/growth regulated oncogene	90.20 ± 38.75	55.46 ± 18.25^*^
***B. Plasma lipids (mg/dL):***		
Total cholesterol (TC)	70.20 ± 12.15	68.90 ± 12.80
Low-density lipoprotein cholesterol (LDL-C)	42.98 ± 8.04	33.18 ± 8.40^*^
High-density lipoprotein cholesterol (HDL-C)	22.70 ± 7.80	32.85 ± 10.67 ^*p* = 0.063^
Triglycerides	60.11 ± 11.21	72.78 ± 18.03
TC:HDL-C ratio	3.71 ± 1.66	2.11 ± 0.59^*^
LDL-C:HDL-C ratio	2.26 ± 1.29	0.50 ± 0.23^**^
***C. Faecal bile acid content (as a ratio of control group):***		
Total bile acids	NA	1.14 ± 0.18 ^*p* = 0.063^
Conjugated bile acids	NA	0.97 ± 0.41
Unconjugated bile acids	NA	1.14 ± 0.19 ^*p* = 0.052^
Conjugate/Unconjugated ratio	NA	0.86 ± 0.38
Cholic acid	NA	1.60 ± 0.40^*^

Data represents the mean ± SD of 6 to 10 rats per group (A) or 10 rats per group (B and C). Values of p were ^*^p < 0.05 or ^**^p < 0.05 or as stated compared to the control group. NA, not applicable.

### Plasma SCFA

**Figure 1:**
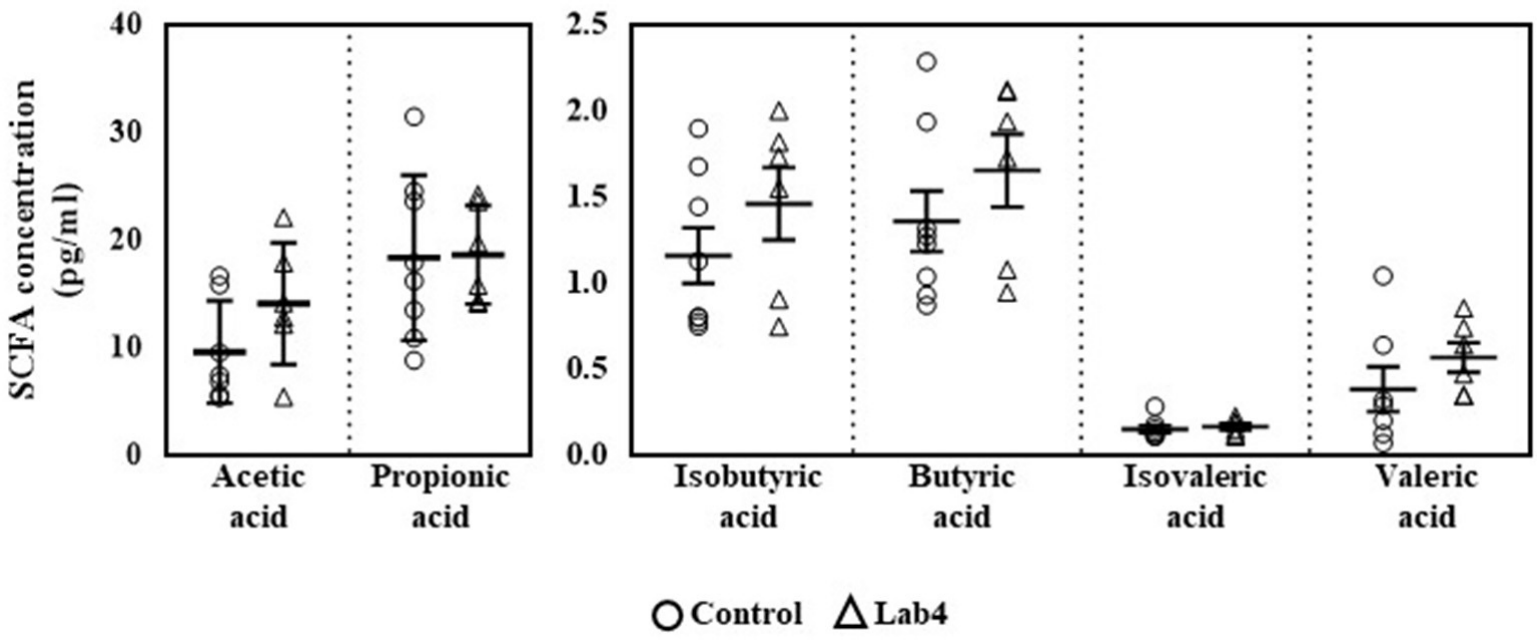
Plasma levels of SCFA in Wistar rats after 90 days supplementation. Plasma levels of SCFA were quantified in control or Lab4 supplemented rats. Data represent the means ± SD of six to eight rats per group. SCFA, short chain fatty acid.

Plasma SCFA concentrations were comparable between groups (Figure 1) although levels in the Lab4 group were consistently higher than in the control group: valeric acid (0.56 ± 0.21 *vs*. 0.38 ± 0.34 pg/ml respectively, 47.4 % increase), acetic acid (14.04 ± 5.64 *vs*. 9.56 ± 4.74 pg/ml respectively, 46.9 % increase), isobutyric acid (1.46 ± 0.51 *vs*. 1.16 ± 0.46 pg/ml respectively, 25.9 % increase), butyric acid (1.65 ± 0.52 *vs*. 1.36 ± 0.50 pg/ml respectively, 21.3 % increase), isovaleric acid (0.16 ± 0.04 *vs*. 0.15 ± 0.59 pg/ml respectively, 6.7 % increase) and propionic acid (18.59 ± 4.57 *vs*. 18.31 ± 7.68 pg/ml respectively, 1.5 % increase).

### Plasma Lipids

Plasma total cholesterol (TC) and triglycerides (TG) were similar between groups after 90 days feeding. LDL-C was significantly lower (−22.8 %, *p* = 0.029, Table 1B) and HDL-C was higher (44.7 %, *p* = 0.063) in the Lab4 group compared to the control. Between group ratios of LDL-C:HDL-C (−50.4 %, *p* = 0.007) and TC:HDL-C (−36.8 %, *p* = 0.028) were significantly lower for the Lab4 group. Bodyweights and food consumption rates did not differ between groups (Supplementary Figure S1).

### Faecal Bile Acid Profiling

**Figure 2:**
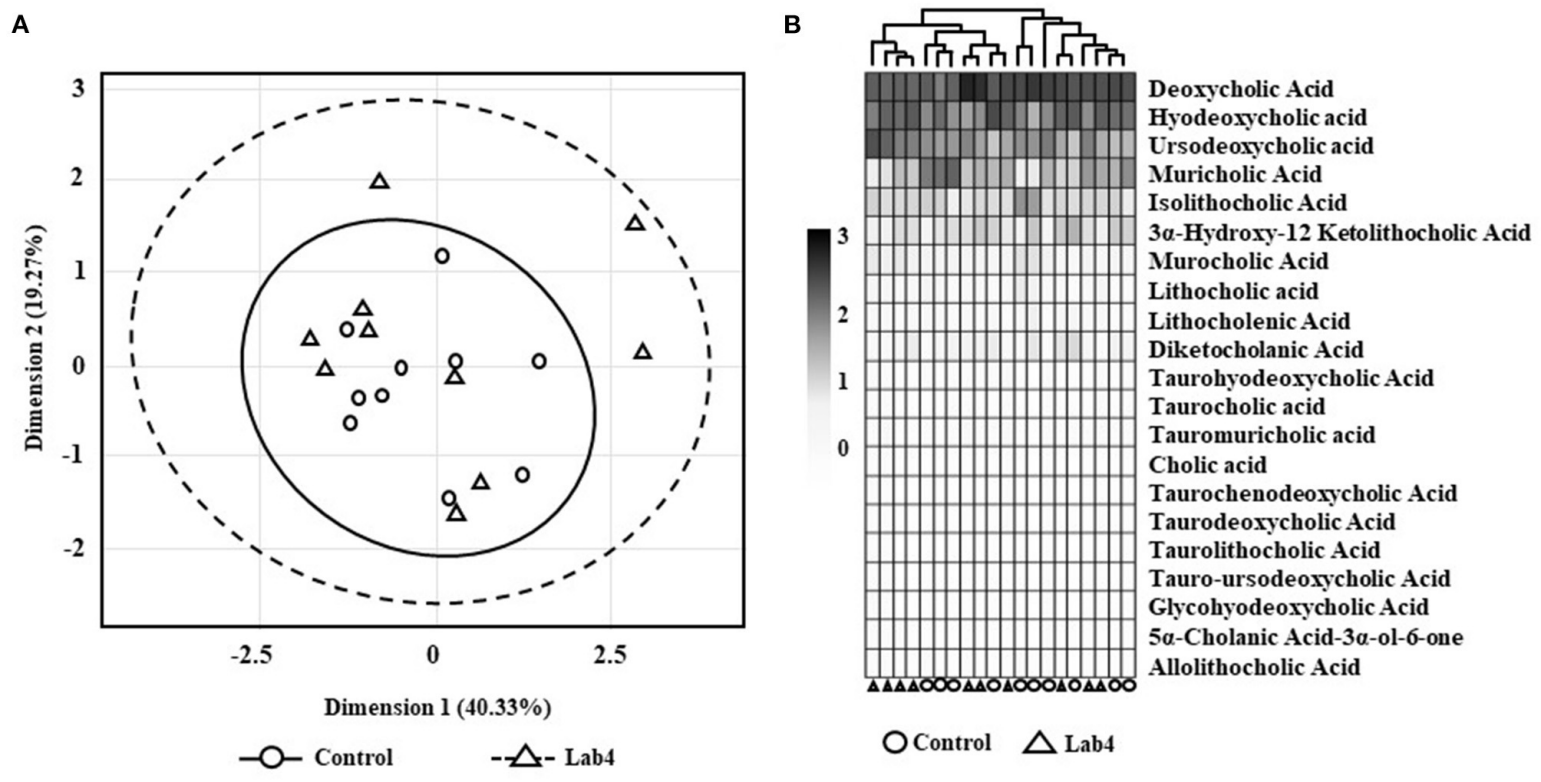
Faecal bile acid profiles of Wistar rats after 90 days supplementation. **(A)** Principle component analysis (PCA) scores plot of bile acid signatures from control or Lab4 supplemented rats or **(B)** heat-map of the bile acid relative intensity for each rat. Analysis was performed on 10 rats per group.

No significant differences were observed between groups in the faecal bile acid profiles whether by Principle Component Analysis (PCA; Figure 2A) or heat-map analysis (Figure 2B) but a more variable spatial organisation was observed for the Lab4 group (Figure 2A). Univariate analysis detected 14 % higher levels of both total and unconjugated bile acids (*p* = 0.063 and *p* = 0.052, respectively, Table 1C) and 60 % higher levels of cholic acid (*p* = 0.043) in the Lab4 group compared to the control. No further significant differences were observed (Supplementary Figure S2) and total bile concentrations in blood plasma were consistent between groups (data not shown).

### Spearman's Ranked Correlation Analysis

**Figure 3:**
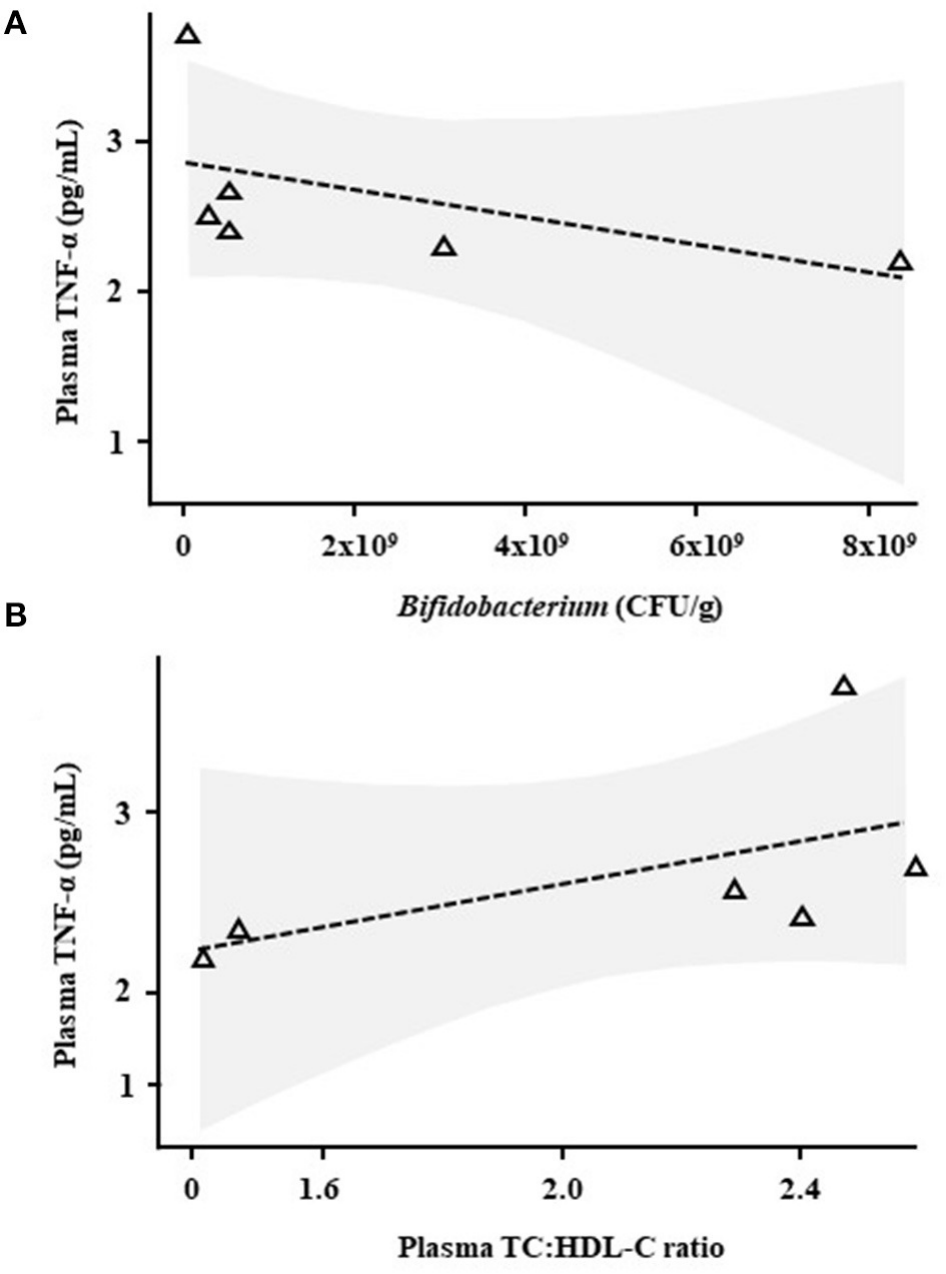
Correlative analysis in Wistar rats after 90 days supplementation with Lab4. Scatter plots between plasma tumour necrosis factor-α (TNF-α) levels and **(A)** faecal *Bifidobacterium* levels or **(B)** the plasma TC:HDL-C ratio in rats receiving Lab4 for 90 days. Analysis was performed on pairwise data from six rats. TNF, tumour necrosis factor; TC, total cholesterol; HDL-C, high density lipoprotein cholesterol.

Correlative analysis was performed for plasma cytokines/chemokines, plasma SCFAs, plasma lipids, faecal bile acids and faecal numbers of viable lactobacilli and bifidobacteria [measured by Baker et al. (13), data shown in Supplementary Figure S3]. TNF-α levels correlated negatively with faecal levels of *Bifidobacterium* (*R* = −0.94, *p* = 0.0048, Figure 3A) and positively with plasma TC:HDL-C (*R* = 0.89, *p* = 0.019, Figure 3B) only in the Lab4 group.

## Discussion

During this 90-day feeding study in wild-type Wistar rats, daily supplementation with the Lab4 probiotic imparted an anti-inflammatory effect by reducing plasma levels of IL-6, TNF-α and KC/GRO and improved plasma cholesterol profiles, possibly *via* the enhancement of bile acid deconjugation in the intestines.

Elevated levels of pro-inflammatory cytokines have been detected in healthy asymptomatic individuals and are thought to be triggered by many factors including stress, poor diet, physical inactivity and lack of sleep (20). Plasma analysis of the Lab4 supplemented rats revealed significant reductions in the pro-inflammatory cytokines IL-6 and TNF-α and the pro-inflammatory chemokine KC/GRO implying an anti-inflammatory capability. These findings align with the outcomes from a previous study where analysis *ex vivo* of blood mononuclear cells collected from Lab4 supplemented healthy adult participants showed reduced secretion of IL-6 and TNF-α (12). Other probiotic studies have demonstrated the ability of bifidobacteria and/or lactobacilli to reduce plasma levels of IL-6 and/or TNF-α in pre-clinical animal models (21–23) and human subjects (24) and *Lactobacillus fermentum* JDFM216 supplementation reduced serum KC/GRO in ageing mice (25). Inflammation is a major risk factor of CVD and a myriad of pro-inflammatory cytokines and chemokines have been associated with this disease progression (26). IL-6 and TNF-α are predominantly produced by cells of the innate immune system including monocytes, macrophages and natural killer cells in response to a physiological challenge (e.g., oxidative stress) (27). Pharmacological inhibition of their activity is considered a promising approach to reduce CVD risk (28, 29).

The anti-inflammatory capabilities of probiotics have been linked, at least in part, with the generation of SCFAs such as acetic, propionic and butyric acid, *via* the fermentation of dietary fibre (30). In rats receiving Lab4, a general pattern of increase was observed for each SCFA measured with notable changes occurring for valeric, acetic and butyric acids. Each strain of the Lab4 consortium possesses gene sequences encoding phosphate acetyltransferase and acetate kinase that are involved in the generation of acetate (31): acetate is recognised as an anti-inflammatory SCFA known to inhibit the expression of IL-6 by colon cells (32). The recent study of Yang et al. showed that administration of *Lactobacillus fermentum* ZJUIDS06 increased levels of acetic, propionic and butyric acid in the colons of hypercholesterolemic hamsters whilst significantly reducing plasma cholesterol levels (33). SCFA are known to have pleiotropic effects on the host and there is growing evidence supporting beneficial effects on cholesterol metabolism (34).

Hypercholesterolemia is a major risk factor for CVD (35) and reductions in circulating cholesterol levels, particularly LDL-C, are encouraged to reduce the risk of CVD (36) even in overtly healthy subjects (8). In this study with Lab4 supplemented rats we showed a 22.8 % reduction in the plasma LDL-C concentration even in the absence of any hypercholesteraemic profile. Such outcomes could imply a potential cardiovascular benefit and expand on the outcomes of our own studies with Lab4 (13, 14, 16, 17) whilst adding to the growing evidence supporting an LDL-C lowering capability by probiotics (7). In contrast, due to their function in the disposal of excess cholesterol *via* the liver, high circulating HDL-C levels are linked to a reduced CVD risk (37). In the probiotic fed rats, plasma HDL-C levels were 44 % higher than in the control group – an outcome consistent with findings observed in a study with Lab4 supplemented atherosclerosis prone mice (16). In both studies, the changes in lipid profiles contributed to significant reductions in the TC:HDL and LDL:HDL ratios in the animals receiving Lab4 compared to the controls. The TC:HDL and LDL:HDL ratios are clinically recognised markers of cardiovascular risk (37).

Reduction of cholesterol by probiotic bacteria can involve the deconjugation of bile acids in the intestinal lumen which is mediated by the actions of inherent BSH enzymes (11). The Lab4 consortium has BSH activity (13, 17) and increased excretion rates of deconjugated bile acids (both total and cholic) were seen with Lab4 treated Wistar rats indicating an ability to impact upon bile acid metabolism during normal metabolic processes and consistent with the reductions observed in circulating LDL-C. The ability of Lab4 to assimilate cholesterol and regulate the transport of cholesterol across the intestinal epithelium (17) may have contributed to its hypocholesterolaemic effects and represents a avenue of future research.

Correlative analyses showed that plasma levels of TNF-α were negatively associated with numbers of faecal bifidobacteria in the Lab4 supplemented rats. As previously mentioned, anti-inflammatory activity is recognised in bifidobacteria (38), the Lab4 consortium comprises *Bifidobacterium bifidum* and *Bifidobacterium animalis* subsp. *lactis* and increased levels of viable bifidobacteria (and lactobacilli) were observed in the faeces of the rats receiving Lab4 (Supplementary Figure S3). In addition, TNF-α levels positively correlated with the plasma TC:HDL-C ratio potentially linking circulating TNF-α and CVD development (39).

There are a number of limitations of the study. Only healthy male rats receiving a single dose of probiotic were included in the analysis. Future studies should assess the cardiovascular and inflammatory impact of different doses of Lab4 in healthy male and female subjects and in cohorts with high risk of cardiovascular disease.

In summary, 90-day supplementation of rats with the Lab4 probiotic resulted in significant reductions in circulating levels of IL-6, TNF-α and KC/GRO and improvements in circulating lipid profiles, possibly mediated by the increased excretion of bile acids. This study highlights daily supplementation with the Lab4 consortium as a potential prophylactic against CVD and has provided support for future human intervention studies.

## Data Availability Statement

The raw data supporting the conclusions of this article will be made available by the authors, without undue reservation.

## Ethics Statement

The animal study was reviewed and approved by the Institutional Ethics Committee of INTOX Pvt. Ltd. (Maharashtra, India) who are a specialist animal testing facility compliant with all local ethical regulations and with the OECD Principles of Good Laboratory Practice [Organisation for Economic Co-operation and Development (OECD), 1998].

## Author Contributions

SFP, TSW, and DRM were responsible for the design of the study. Experiments were performed by TSW, LMB, and AAJ. Data and statistical analyses were performed by GM, DRM, and TSW. TRH, JRM, GR, and PDF provided assistance and knowledge that was vital to the completion of the manuscript. DRM, JD, and SFP prepared the manuscript. All authors contributed to the review of the manuscript.

## Funding

JRM and the Department of Metabolism, Digestion, and Reproduction at Imperial College London received funding from the National Institute of Health Research (NIHR), the Biomedical Research Centre (BRC) based at Imperial College London, and Imperial College Healthcare NHS Trust.

## Conflict of Interest

TSW, GM, JD, AAJ, SFP, and DRM were employed by the company Cultech Limited. The remaining authors declare that the research was conducted in the absence of any commercial or financial relationships that could be construed as a potential conflict of interest.

## Publisher's Note

All claims expressed in this article are solely those of the authors and do not necessarily represent those of their affiliated organizations, or those of the publisher, the editors and the reviewers. Any product that may be evaluated in this article, or claim that may be made by its manufacturer, is not guaranteed or endorsed by the publisher.
