# Supplementary material for: The Impact of Lab4 Probiotic Supplementation in a 90-Day Study in Wistar Rats
Source: Front Nutr. 2021 Nov 25;8:778289. doi: 10.3389/fnut.2021.778289 (PMC8656110; doi:10.3389/fnut.2021.778289)
Supplement: Supplementary file 1 [file Data_Sheet_1.docx]

**Supplementary information: The Impact of Lab4 Probiotic Supplementation in a 90-day Study in Wistar Rats**

**A**

**B**

**Supplementary Figure S1. Changes in (A) body weight and (B) average food consumption of Wistar rats rats after 90 days supplementation with Lab4.** Data represents the mean±standard deviation (SD) of 10 rats per group.

**A**

**B**

**B**

| Bile acid | **Control** | |  | **Lab4** | |
| --- | --- | --- | --- | --- | --- |
|  | **Mean** | **SD** |  | **Mean** | **SD** |
| Lithocholenic acid | 1.00 | 0.50 |  | 1.27 | 1.17 |
| Allolithocholic acid | 1.00 | 0.57 |  | 1.28 | 1.07 |
| Lithocholic acid | 1.00 | 0.31 |  | 1.40 | 1.21 |
| Isolithocholic acid | 1.00 | 0.23 |  | 1.27 | 0.70 |
| Diketocholanic acid | 1.00 | 0.81 |  | 1.50 | 1.21 |
| 3α-Hydroxy-12 Ketolithocholic acid | 1.00 | 0.43 |  | 1.32 | 0.73 |
| 5α -Cholanic Acid-3α -ol-6-one | 1.00 | 0.70 |  | 0.92 | 0.68 |
| Ursodeoxycholic acid | 1.00 | 0.24 |  | 0.93 | 0.31 |
| Murocholic acid | 1.00 | 0.40 |  | 1.27 | 0.76 |
| Deoxycholic acid | 1.00 | 0.13 |  | 1.11 | 0.26 |
| Hyodeoxycholic acid | 1.00 | 0.20 |  | 1.06 | 0.22 |
| Cholic acid | 1.00 | 0.57 |  | 1.50 | 0.50* |
| Muricholic acid | 1.00 | 0.42 |  | 1.38 | 0.60 |
| Glycohyodeoxycholic acid | 1.00 | 0.43 |  | 1.76 | 1.30 |
| Taurolithocholic acid | 1.00 | 0.39 |  | 0.83 | 0.71 |
| Taurochenodeoxycholic acid | 1.00 | 0.37 |  | 1.02 | 0.29 |
| Taurohyodeoxycholic acid | 1.00 | 0.41 |  | 0.88 | 0.39 |
| Tauro-ursodeoxycholic acid | 1.00 | 0.37 |  | 1.04 | 0.40 |
| Taurodeoxycholic Acid | 1.00 | 0.50 |  | 1.10 | 0.72 |
| Taurocholic acid | 1.00 | 0.39 |  | 0.98 | 0.49 |
| Tauromuricholic acid | 1.00 | 0.38 |  | 0.96 | 0.45 |

**Supplementary Figure S2. Bile acid profiles.** Relative intensities of faecal bile acid signatures gathered from ultra-performance liquid chromatography-mass spectrometry (UPLC-MS) analysis for control (white bars) and Lab4 (black bars) groups (Panel A) and the ratio of faecal bile acid content compared to the control group (Panel B). Data represent the means ± SD of 10 rats per group. ^∗^*p*<0.05 compared to the control group. Data is corrected to dry weight of stool.

**A**

**B**

**Supplementary Figure S3. Faecal counts of viable lactobacilli and bifidobacteria of Wistar rats rats after 90 days supplementation with Lab4** **(adapted from Baker *et al*(1)).** Viable bacterial cell numbers of (A) lactobacilli and (B) bifidobacteria in the faeces of male Wistar rats supplemented for 90 days with Lab4. Data is present as mean±standard deviation (SD) and values of *p* were determined using the Kruskal Wallis one-way analysis of variance on ranks followed by Dunns’s test with bonferroni correction where **p*<0.05 and ** *p*<0.01.

**Supplmentary references**

1. Baker LM, Davies TS, Masetti G, Hughes TR, Marchesi JR, Jack AA, et al. A genome guided evaluation of the Lab4 probiotic consortium. Genomics. 2021.
